# Pediatric reference values of anterior visual pathway structures measured with axis-correction on high-resolution 3D T2 fast spin echo sequences

**DOI:** 10.1186/s12887-022-03637-z

**Published:** 2022-10-08

**Authors:** Stefan Markart, Simon Wildermuth, Johannes Geiss, Erik P. Willems, Veit Sturm, Michael Ditchfield, Stephan Waelti

**Affiliations:** 1 Department of Radiology and Nuclear Medicine, Children’s Hospital of Eastern Switzerland, St. Gallen, Switzerland; 2 Department of Radiology and Nuclear Medicine, Cantonal Hospital St. Gallen, Rorschacher Strasse 95, 9007 St. Gallen, Switzerland; 3 Clinical Trials Unit, Biostatistics, Cantonal Hospital St. Gallen, St. Gallen, Switzerland; 4 Department of Ophthalmology, Cantonal Hospital St. Gallen, St. Gallen, Switzerland; 5 Department of Diagnostic Imaging, Monash Health, Monash Children’s Hospital, Clayton, Australia

**Keywords:** Anterior visual pathway (AVP), Pediatric MRI, High resolution 3D MRI, 3D T2 fast spin echo (FSE) sequence, Multiplanar reformation (MPR)

## Abstract

**Background:**

The size of the anterior visual pathway (AVP) structures is affected by patient age and pathology. Normative data is useful when determining whether pathology is present. AVP structures do not respect the standard planes of magnetic resonance (MR) imaging. The aim of this study was to produce normative age-related and axis-corrected data of the AVP structures using multiplanar reformation (MPR) of high-resolution 3D T2-weighted fast spin echo (3D T2w FSE) images.

**Methods:**

For each patient 32 measurements of AVP structures were obtained in 145 children (2 months - 18 years) with normal brain MR studies on high-resolution 3D T2w FSE images adjusted to the axis of each AVP structure. Descriptive statistics were calculated for different age classes and growth models were fitted to the data and assessed for their performance to create a formal statistical model that allows inference beyond the sample.

**Results:**

Descriptive statistics were compiled in a reference table and prediction plots in relation to age, height, and body surface area (BSA) were obtained from the best overall performing statistical model, also taking field strength (1.5 vs. 3 T) into account. Intraclass correlation coefficient values were calculated for all variables ranging from 0.474 to 0.967, the most reliable being the transverse diameter of the globe, the maximum diameter of the retrobulbar nerve sheath, the intracranial segment of the optic nerve and the transverse diameter of the chiasm. The maximum retrobulbar diameter of the optic nerve sheath and the lateral superoinferior diameter of the chiasm showed no statistically significant change with age.

**Conclusion:**

Detailed charts of reference values for AVP structures as well as prediction plots in relation to age, height and BSA were established using axis-corrected measurements from the MPR of high-resolution 3D T2w FSE images. Furthermore, an Excel spreadsheet that allows users to calculate normative values for the 9 AVP structures of key interest is provided as supplementary material.

**Supplementary Information:**

The online version contains supplementary material available at 10.1186/s12887-022-03637-z.

## Introduction

The anterior visual pathway (AVP) includes the globe with its lens, the optic nerve with its sheath, and the optic chiasm. The optic nerve begins at the optic disc and ends at the optic chiasm. In between, it can be divided into intraorbital, intracanalicular and intracranial segments. The optic nerve is surrounded by pia mater, arachnoid mater, and dura mater (commonly referred to as the “nerve sheath”) as well as cerebrospinal fluid (CSF) in the subarachnoid space. The sheath encases the optic nerve from the globe to the intracranial foramen of the optic canal (enveloping the intraorbital and intracanalicular segments) and is absent in the intracranial segment and the optic chiasm [1].

Various congenital and acquired pathologies can affect the size of AVP structures in children and adolescents. Enlargement of the optic nerves occurs in conditions such as optic nerve glioma, (e.g., in Neurofibromatosis type 1), Krabbe’s disease or Leber hereditary optic neuropathy and small optic nerves can be seen in septo-optic dysplasia or hereditary optic nerve atrophies [2–4]. Enlargement of the optic nerve sheath is a hallmark of idiopathic intracranial hypertension [5]. Magnetic resonance imaging (MRI) is commonly used to image these pathologies [6, 7].

Normative MRI values of AVP structures can be found in the literature, however few of them include children and adolescents [5, 6, 8, 9]. Only two studies, both in adults, have used 3D T2-weighted FSE (3D T2w FSE) sequences to adjust the image plane along the course of the respective portion of the AVP to produce more accurate and reproducible measurements of each structure, not limited to the three standard image planes [10, 11].

The aim of this study was to define a normative range of dimensions of axis-corrected AVP structures for all pediatric age groups (0–18 years) in relation to age, height and body surface area (BSA) using a high-resolution 3D T2w FSE MR sequence, and to produce predictions from formal statistical models that quantify and visualize structure-specific growth curves as well as their association with height and BSA.

## Materials and methods

### Patient selection

This prospective study was carried out in a single children’s hospital and was approved by the local ethics committee as well as conducted in compliance with ICH-GCP-rules and the Declaration of Helsinki. Between May 2020 and April 2021 two pediatric radiologists selected all patients who were likely to have normal head MRI scans based on the clinical information of the referrals. Typical indications in which normal findings are often found include seizures, developmental delay, headaches and migraines, tumor anxiety, psychosis, and mild traumatic brain injury. Children with a known history of diseases involving the head (including the brain, CSF space, orbit, and skull) or known systemic/syndromic disorders were excluded. Informed consent was obtained for the remaining patients, signed either by the patients themselves (above the age of 14) or their parents (below the age of 14). In these patients, a high-resolution 3D T2w FSE MR sequence of the globes, optic nerves and optic chiasm was acquired in addition to the sequences necessary to answer the referring physician’s questions. All MRI studies were interpreted by pediatric radiologists or neuroradiologists. Examinations that, contrary to expectation, revealed any pathologic findings were excluded. A total of 145 normal studies of 145 children were included. Demographic data of all participants were noted, including age, gender, height and weight.

### MRI examination

Examinations were performed on 3 T or 1.5 T scanners (Siemens Healthcare, Erlangen, Germany) using a 64-channel head/neck coil in 3 T scanners and a 20-channel head/neck coil in 1.5 T scanners. The scan volume of the axial 3D T2w FSE sequence included both globes, both optic nerves and the entire optic chiasm. Detailed parameters of the axial 3D T2w FSE sequence are given in Table 1. Patients up to about 6 years of age were examined under sedation or anesthesia, performed by pediatric anesthetists.

**Table 1:** Scan parameters

Variable	Field strength	
	1.5 T	3 T
Repetition rime (TR)	1400 ms	1300 ms
Echo time (TE)	181 ms	144 ms
Flip angle	150 °	120 °
Slice thickness	0.6 mm	0.5 mm
Matrix size	256 × 256	320 × 336
Field of view	150 × 150 mm	157 × 150 mm
Acquisition time	4 min 51 sec	4 min 37 sec

Scan parameters of the 3D fast spin-echo (FSE) sequence in 1.5 T and 3 T scanners

### Image analysis and measurements

All measurements and subjective evaluations were performed independently by two pediatric radiologists with an overall radiological experience of 11 years (StW) and 7 years (SM). Measurements were performed using the distance measurement tool within the picture archiving and communication system (PACS; Dedalus DeepUnity Diagnost 1.1.0.1, Germany). The measurement accuracy was one digit after the decimal point.

Patients were reviewed in a random order and both readers were blinded to patient data. For every patient, each side was evaluated separately. Whenever one of the two readers deemed the measurement of a diameter at a predefined position only to be possible at an insufficient accuracy (e.g., due to motion artefact), the second radiologist’s measurement at the same position was discarded.

**Fig. 1:**
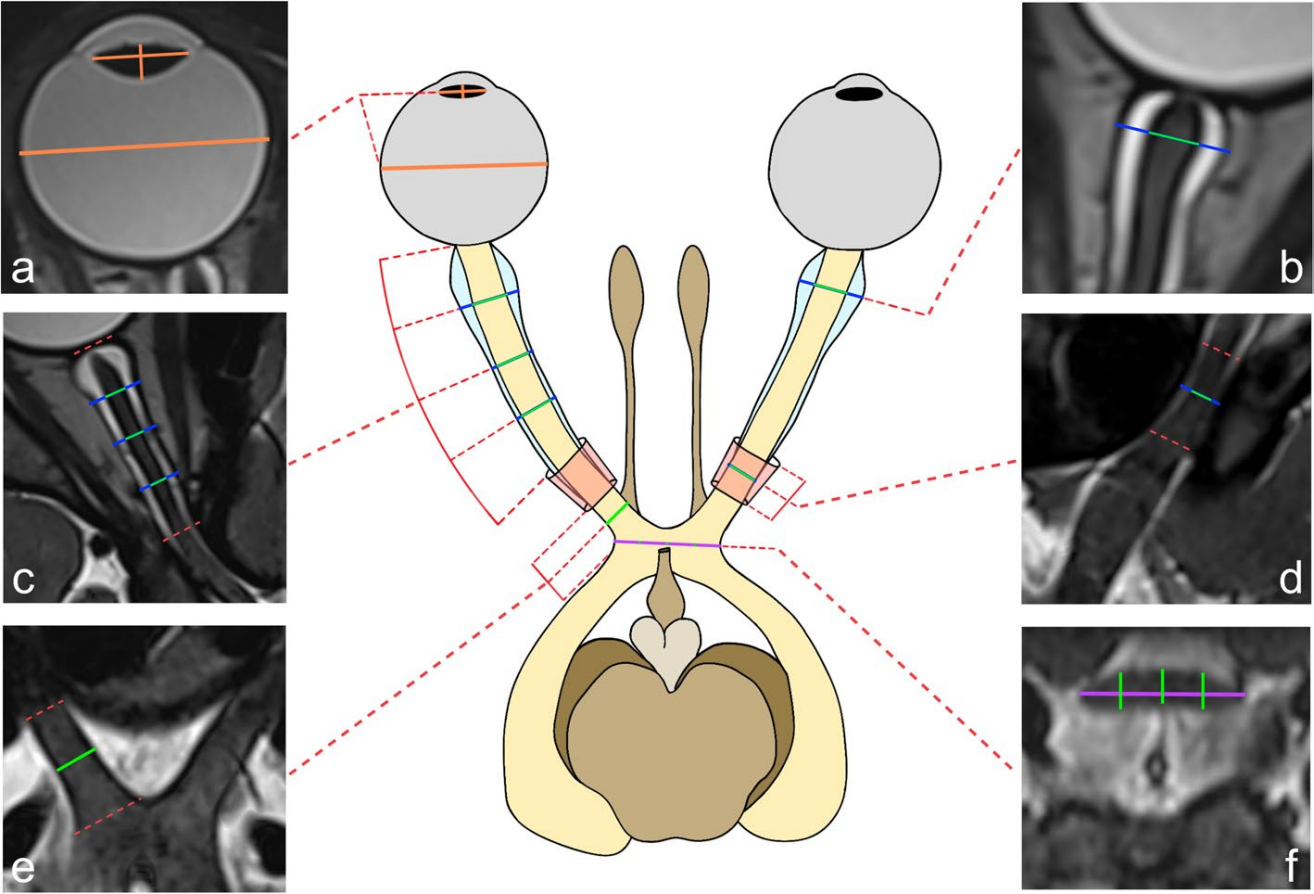
Schematic drawing and corresponding axis-corrected MR images of the different parts of the anterior visual pathway with diameter measurements of the globe and lens (**a**), of the retrobulbar optic nerve and nerve sheath (**b**), of the intraorbital optic nerve and nerve sheath segment (**c**), of the intracanalicular optic nerve and nerve sheath segment (**d**) and of the intracranial optic nerve segment (**e**), all of them measured on individually reformatted axial planes. Diameter measurements of the optic chiasm were obtained on a reformatted coronal plane (**f**)

**Fig. 2:**
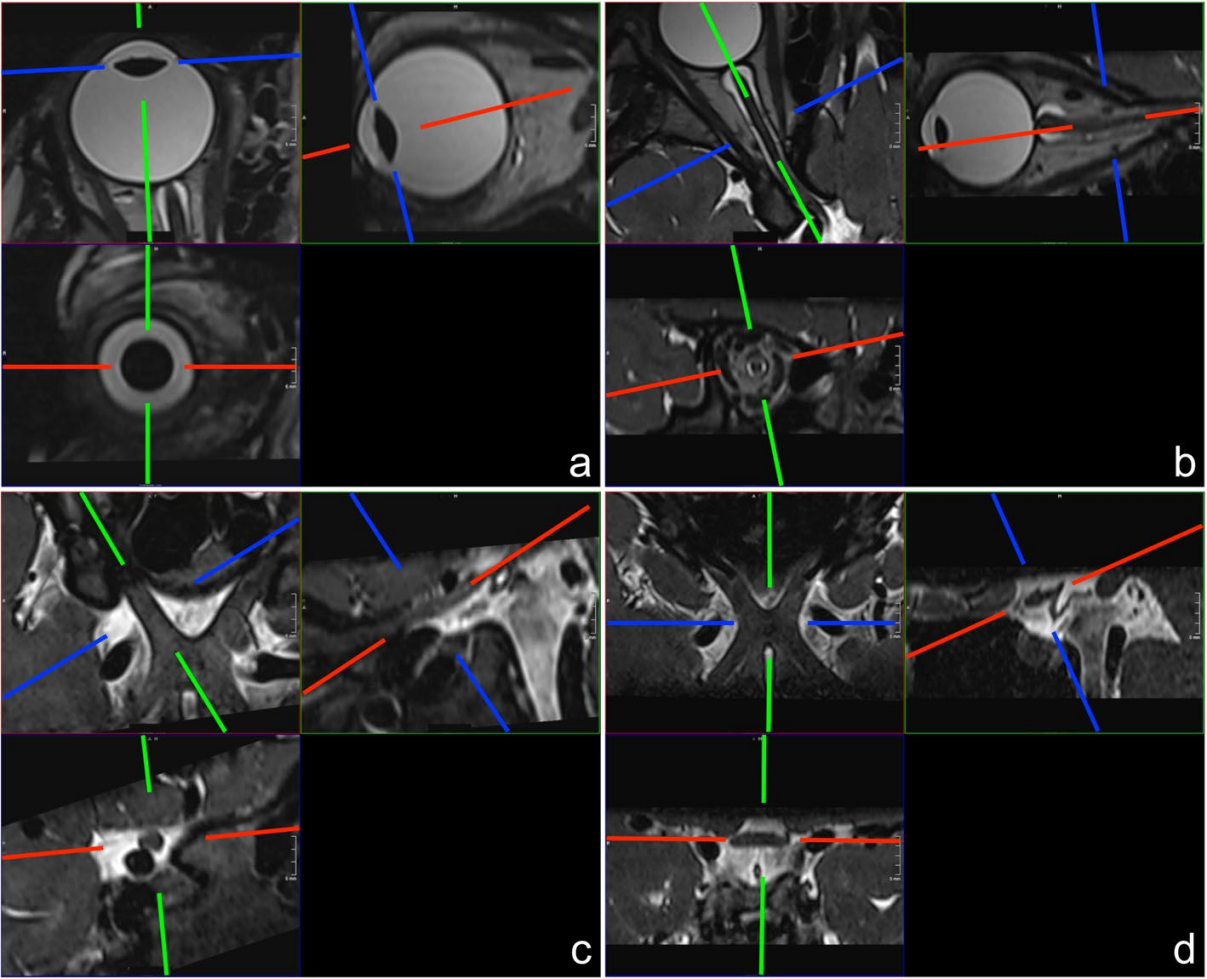
Sample images of axis-corrected multiplanar reconstructions of the lens (**a**), the intraorbital/intracanalicular nerve (**b**), the intracranial optic nerve (**c**) and the optic chiasm (**d**)

A total of 32 measurements were performed per patient, consisting of 15 measurements on the left, 15 measurements on the right, and 2 measurements in the middle (transverse chiasm and superoinferior median), as shown in Fig. 1. All measurements were obtained on images reconstructed using multiplanar reformation (MPR) longitudinal and axial to each structure as illustrated in Fig. 2.

At the level of the globe, maximum transverse and anteroposterior diameters of the lens were obtained by reformatting the images along the axes of the lens using MPR (Fig. 1a). Using the same reformation, the maximum transverse diameter of the globe was obtained by measuring between the inner borders of the globe wall, parallel to the transverse diameter of the lens (Fig. 1a).

**Fig. 3:**
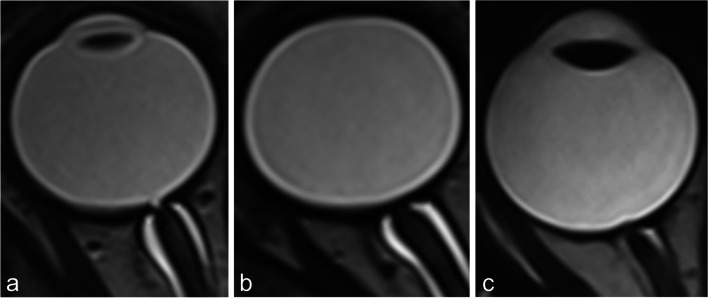
Three types of optic disc morphology: excavation (**a**), flat (**b**) and protrusion (**c**)

In addition, the morphology of the optic disc was assessed subjectively and scored as either a flat disc, a optic disc excavation, or protrusion of the disc (Fig. 3).

Measurements of the optic nerve diameters were obtained at 6 different positions and of the optic nerve sheath at five different positions, always using MPR to reformat the axial images along the longitudinal axis of the nerve to account for its tortuosity. Nerve sheath diameters were measured between the outer borders of the sheath, optic nerve diameters were measured between both nerve borders.

Both, nerve and nerve sheath diameters, were measured at the level of the maximum width of the retrobulbar nerve sheath (Fig. 1b), at 1/4 (position 1 out of 3), 2/4 (position 2 out of 3) and 3/4 (position 3 out of 3) of the intraorbital nerve length (Fig. 1c) and at 1/2 of the optic canal length (Fig. 1d). The diameter of the intracranial nerve segment was measured at 1/2 of its length (Fig. 1e). In addition, the tortuosity of the optic nerve was assessed subjectively and scored as either straight or tortuous.

The measurements of the optic chiasm diameters were obtained on a reformatted coronal slice perpendicular to the longitudinal axis of the chiasm, which can vary greatly in steepness. The maximum transverse diameter of the chiasm was obtained. The transverse diameter was then quartered and superoinferior diameters of the optic chiasm were determined at the level of the resulting three points (Fig. 1f).

### Statistical analysis

To produce the reference table and statistical models, bilateral variables were averaged across the two sides and data was not analyzed separately for the two genders, given that neither side- nor gender-differences were clinically expected, or statistically apparent in a preliminary evaluation of the first 40 patients. The table of reference values was restricted to 9 continuous variables, capturing 4 key neuro-anatomical structures of interest, and taking both location and Inter-Reader-Reliability of the measurement into account (bold structures in Table 2). In addition, the two categorical variables (shape of the fovea: flat, excavation, protrusion; and tortuosity of the optic nerve: absent, present) were also examined.

**Table 2:** Intraclass correlation coefficients for 17 continuous variables of AVP structures

Structure	Variable	n		ICC		95%-CI	
		Left	Right	Left	Right	Left	Right
Eye	**Lens trans**	**112**	**118**	**0.689**	**0.804**	**0.610–0.755**	**0.749–0.847**
	Lens antpost	123	130	0.652	0.736	0.566–0.724	0.666–0.793
	**Globe trans**	**140**	**140**	**0.952**	**0.967**	**0.937–0.963**	**0.957–0.975**
Nerve	**Nerve retrobulbar**	**124**	**125**	**0.601**	**0.602**	**0.505–0.681**	**0.508–0.683**
	Nerve 1/3	118	119	0.581	0.568	0.482–0.665	0.467–0.653
	Nerve 2/3	121	120	0.686	0.696	0.606–0.752	0.618–0.761
	**Nerve 3/3**	**133**	**125**	**0.673**	**0.734**	**0.591–0.741**	**0.664–0.791**
	Nerve canalis opticus	135	132	0.474	0.542	0.361–0.574	0.438–0.632
	**Nerve prechiasmatic**	**136**	**134**	**0.807**	**0.828**	**0.754–0.850**	**0.779–0.867**
Sheath	**Sheath retrobulbar**	**125**	**125**	**0.771**	**0.874**	**0.709–0.821**	**0.838–0.903**
	Sheath 1/3	117	120	0.709	0.666	0.633–0.771	0.582–0.736
	Sheath 2/3	119	121	0.758	0.794	0.694–0.811	0.737–0.839
	**Sheath 3/3**	**132**	**127**	**0.747**	**0.697**	**0.680–0.802**	**0.619–0.761**
	Sheath canalis opticus	136	132	0.659	0.728	0.574–0.730	0.657–0.786
Chiasm	**Supinf lat**	**112**	**112**	**0.729**	**0.731**	**0.658–0.788**	**0.660–0.789**
	Supinf middle	112		0.642		0.554–0.716	
	**Trans**	**117**		**0.948**		**0.932–0.960**	

Intraclass Correlation Coefficients for 17 continuous variables measured from high resolution, axis-adjusted MRI-imagery on a total of 145 patients, by 2 independent readers (*n* Number of patients, *ICC* Intraclass Correlation Coefficient, *CI* Confidence Interval). Variables of primary interest are highlighted in a bold typeset

*Abbreviations*: *trans* Transverse, *antpost* Anteroposterior, *1/3* Position 1 out of 3, *2/3* Position 2 out of 3, *3/3* Position 3 out of 3, *supinf* Superoinferior, *lat* Lateral

#### Inter-reader-reliability

The level and consistency of agreement between the two readers was assessed by calculating Intraclass Correlation Coefficients (ICC) for all continuous, and Cohen’s κ for all categorical variables, using the “psych” package in R version 4.0 [12, 13].

#### Age-specific reference table and statistical growth models

After establishing that Inter-Reader-Reliability on all variables was sufficiently high, consensus values were calculated as the mean of the independent measurements taken by the 2 readers, and for bilateral variables (i.e., all, except for the transverse diameter of the optic chiasm), subsequently taking the inpatient mean of their left and right side. Descriptive statistics to characterize the mode and dispersion of the 9 primary outcome variables were calculated for the different age-classes and compiled in a reference table. To complement this table with predictions from a formal statistical model to allow inference beyond the sample and to take MRI field strength into account, different growth models were fitted to the data and assessed for their performance. Four commonly used growth curves to quantify organismal development [14] were investigated: two linear models (in which the diameter of each outcome was expressed as a function of age and MRI field strength [a + b_1_ × Age + b_2_ × MRI field strength, or the natural log of age [a + b_1_ × ln(Age) + b_2_ × MRI field strength]), and two non-linear models (expressing each outcome as a function of age using the Monod [(a × Age)/(b + Age) + c × MRI field strength] or von Bertalanffy [a × (1 - e^-b × Age + c × MRI field strength^)] equation). To identify the most appropriate approach given the data at hand, all models were compared using the Akaike Information Criterion (AIC [15];). Prediction plots of the preferred model equation were generated using the “ggeffects” package [16] in R version 4.0. Similar plots and model predictions were produced to showcase the association between the 9 key variables of interest and patient height as well as BSA. In addition to prediction plots, which allow a quick visual assessment of normative values, we also provide an “interactive” spreadsheet with which users can calculate normative values themselves, for any given patient age or height and selecting the field strength of the MRI scanner they have at their disposal (Supplementary Material).

## Results

### Description of the data sample

Between May 2020 and April 2021, the following number of head MRI examinations was performed in children within the respective age groups:

- 0–3 years: a total of 116 MRI; 103 pathological (89%) and 13 normal (11%)
- 4–6 years: a total of 81 MRI; 64 pathological (79%) and 17 normal (21%)
- 7–9 years: a total of 102 MRI; 83 pathological (81%) and 19 normal (19%)
- 10–12 years: a total of 87 MRI; 68 pathological (78%) and 19 normal (22%)
- 13–15 years: a total of 108 MRI; 80 pathological (74%) and 28 normal (26%)
- 16–18 years: a total of 190 MRI; 141 pathological (75%) and 49 normal (25%)

After excluding all pathological MR examinations, the study sample was 145 patients (75 girls, 70 boys), aged between 77 days and 18.8 years. Descriptive statistics in terms of age, height and weight are presented in Table 3. 83% (*n* = 120; median age = 13.00 years, IQR = 7.12–16.14 years) of patients were examined in 3 T scanners, the remaining 17% (*n* = 25, median age = 16.56 years, IQR = 12.35–17.27 years) were examined in 1.5 T scanners.

**Table 3:** Descriptive statistics characterising the different age groups in our sample of 145 patients, in terms of age, height and weight

Variable	Age group							
	0–3				4–6			
	**n**	**mean**	**sd**	**95%-CI**	**n**	**mean**	**sd**	**95%-CI**
Age (years)	13	2.49	1.19	0.15–4.82	17	5.23	0.79	3.67–6.79
Height (m)	13	0.90	0.14	0.63–1.17	17	1.11	0.08	0.96–1.26
Weight (kg)	13	13.54	4.00	5.69–21.38	17	20.69	6.88	7.21–34.18
BSA (m^2^)	13	0.57	0.13	0.31–0.83	17	0.79	0.13	0.52–1.05
	7–9				10–12			
	**n**	**mean**	**sd**	**95%-CI**	**n**	**mean**	**sd**	**95%-CI**
Age (years)	19	8.59	1.01	6.62–10.56	19	11.62	0.93	9.81–13.44
Height (m)	19	1.31	0.08	1.15–1.47	19	1.46	0.11	1.24–1.68
Weight (kg)	19	29.59	6.57	16.71–42.46	19	39.55	10.69	18.61–60.50
BSA (m^2^)	19	1.03	0.13	0.78–1.28	19	1.27	0.19	0.89–1.64
	13–15				16–18			
	**n**	**mean**	**sd**	**95%-CI**	**n**	**mean**	**sd**	**95%-CI**
Age (years)	28	14.61	0.82	13.01–16.21	49	17.2	0.8	15.58–18.86
Height (m)	28	1.68	0.08	1.51–1.84	49	1.7	0.1	1.52–1.90
Weight (kg)	28	59.50	11.90	36.18–82.82	49	66.7	14.3	38.62–94.70
BSA (m^2^)	28	1.67	0.19	1.29–2.04	49	1.78	0.21	1.37–2.18

*n* Sample size, *sd* Standard deviation, *BSA* Body surface area, *CI* Confidence Interval

### Inter-reader-reliability

Table 2 presents the ICC-values for all 17 continuous variables, with all but the last two measured on both the left and right side of the patient. Calculated values ranged from 0.474 to 0.967, while for the 9 variables of primary interest, the range was 0.601–0.967, the latter representing a “good to excellent” level of agreement.

Inter-Reader-Reliability for the two categorical variables was expressed by calculating Cohen’s κ. A subjective reading of these values suggests an “excellent” level of agreement between readers on the shape of the optic disc (left: 0.794 [95%-CI = 0.673–0.915], right: 0.836 [95%-CI = 0.726–0.945]), whereas agreement on tortuosity of the optic nerve was “fair to good” (left: 0.675 [95%-CI = 0.477–0.873], right: 0.575 [95%-CI = 0.325–0.825]).

### Reference table, prediction plots and spreadsheet

Table 4 presents the age-specific normative range of the 9 continuous key variables of interest. Reported numbers represent consensus scores, calculated as the mean across independent measurements taken by the two readers, and for bilateral variables (i.e., all, except for the transverse diameter of the optic chiasm), subsequently taking the inpatient mean of their left and right side. Age groups were defined in whole years so that, for example, a 3.7-year-old patient was assigned to the 0–3 years cohort.

**Table 4:** Age-specific modal values of 9 key variables of interest

Variable	Age group							
	0–3				4–6			
	**n**	**mean**	**sd**	**95%-CI**	**n**	**mean**	**sd**	**95%-CI**
Lens trans	12	8.41	0.39	7.65–9.18	14	8.76	0.27	8.24–9.29
Globe trans	13	21.61	0.86	20.84–22.38	16	22.58	0.83	22.05–23.10
Nerve retrobulbar	12	2.86	0.31	2.09–3.62	16	2.99	0.29	2.46–3.51
Nerve 3/3	11	2.16	0.16	1.40–2.93	17	2.46	0.29	1.94–2.99
Nerve prechiasmatic	13	3.51	0.42	2.75–4.28	17	3.75	0.28	3.23–4.28
Sheath retrobulbar	12	6.42	0.48	5.65–7.18	16	6.65	0.72	6.12–7.17
Sheath 3/3	12	3.98	0.61	3.21–4.74	17	4.19	0.64	3.67–4.72
Chiasm supinf lat	9	2.67	0.40	1.90–3.43	14	2.63	0.27	2.11–3.16
Chiasm trans	9	11.12	1.10	10.35–11.88	15	11.69	1.45	11.16–12.21
	7–9				10–12			
	**n**	**mean**	**sd**	**95%-CI**	**n**	**mean**	**sd**	**95%-CI**
Lens trans	16	8.59	0.35	7.90–9.28	14	9.03	0.26	8.53–9.53
Globe trans	18	22.58	0.65	21.90–23.27	19	23.27	1.01	22.77–23.78
Nerve retrobulbar	16	3.14	0.31	2.45–3.82	13	3.19	0.27	2.69–3.69
Nerve 3/3	17	2.56	0.26	1.87–3.24	13	2.66	0.31	2.16–3.16
Nerve prechiasmatic	19	4.02	0.40	3.33–4.70	15	4.21	0.42	3.71–4.71
Sheath retrobulbar	16	6.60	0.98	5.91–7.28	13	6.81	0.70	6.30–7.31
Sheath 3/3	17	4.34	0.56	3.65–5.03	14	4.16	0.59	3.66–4.66
Chiasm supinf lat	16	2.77	0.31	2.08–3.45	12	2.99	0.34	2.49–3.49
Chiasm trans	17	11.83	1.09	11.14–12.52	13	12.85	1.36	12.35–13.35
	13–15				16–18			
	**n**	**mean**	**sd**	**95%-CI**	**n**	**mean**	**sd**	**95%-CI**
Lens trans	22	8.98	0.37	8.25–9.71	34	9.20	0.33	8.56–9.84
Globe trans	27	23.41	0.70	22.68–24.15	47	23.88	1.08	23.24–24.52
Nerve retrobulbar	25	3.20	0.29	2.47–3.93	35	3.22	0.31	2.58–3.86
Nerve 3/3	24	2.60	0.19	1.86–3.33	41	2.69	0.40	2.05–3.33
Nerve prechiasmatic	24	4.22	0.42	3.49–4.95	46	4.36	0.42	3.72–5.00
Sheath retrobulbar	25	6.80	0.70	6.07–7.54	36	6.62	0.66	5.98–7.26
Sheath 3/3	24	4.39	0.51	3.66–5.12	40	4.47	0.49	3.83–5.11
Chiasm supinf lat	21	2.90	0.32	2.17–3.63	40	2.80	0.36	2.16–3.44
Chiasm trans	21	13.03	1.67	12.30–13.76	42	13.67	1.27	13.03–14.31

The unit of measurement of all variables was millimeters (mm), with a measurement accuracy of 1 decimal. Reported numbers represent consensus scores, calculated as the mean across independent measurements taken by 2 readers, and for bilateral variables (i.e., all, except Chiasm trans), subsequently taking the inpatient mean of their left and right side. Note that in the supplementary Materials we provide an ‘interactive’ worksheet that allows users to calculate normative values for each of the 9 AVP structures themselves, for any given patient age or height, and taking the field strength of the MRI scanner into account (*n* Number of patients, *sd* Standard deviation, *CI* Confidence Interval)

*Abbreviations*: *trans* Transverse, *3/3* Position 3 out of 3, *supinf* Superoinferior, *lat* Lateral

**Fig. 4:**
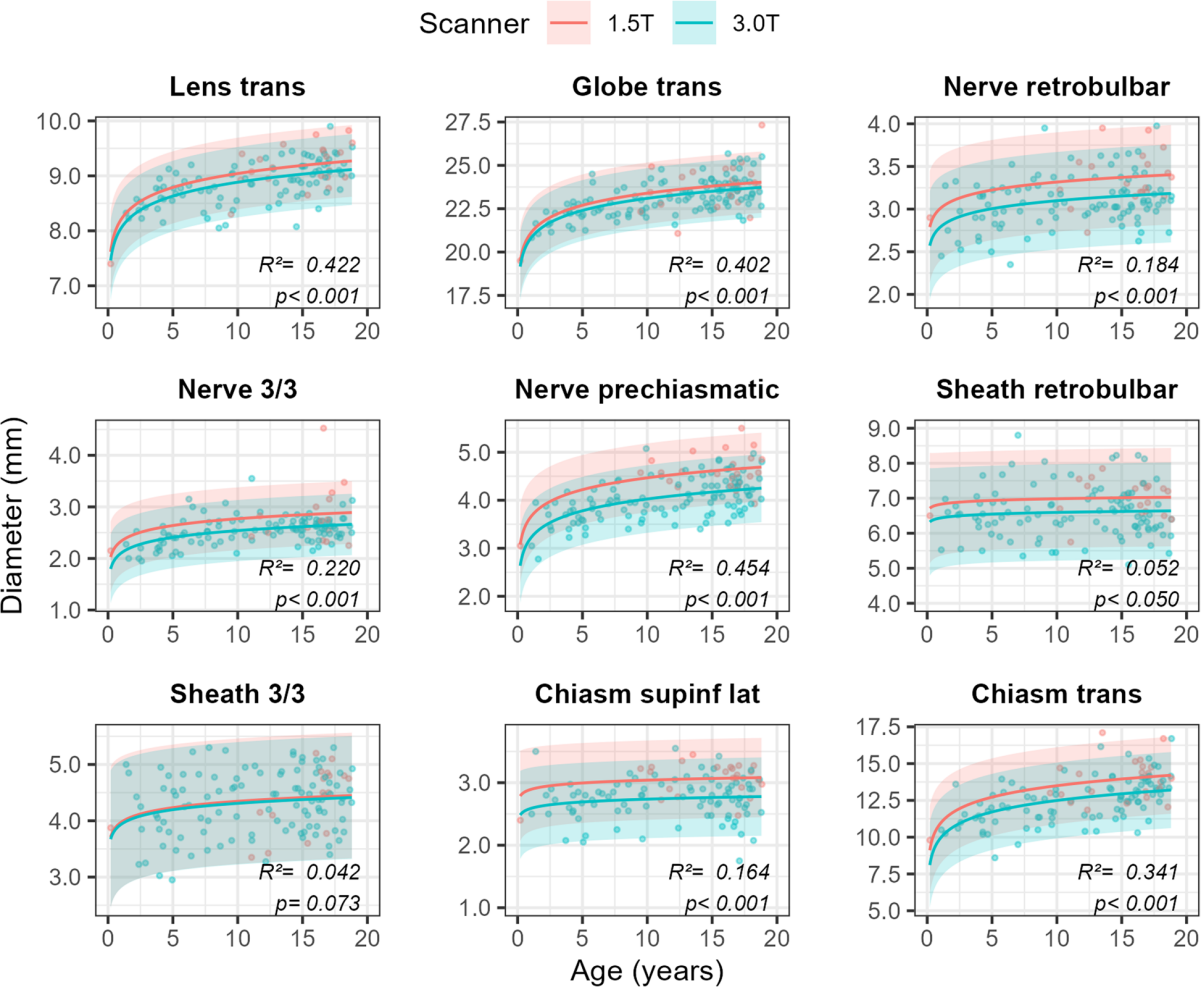
Prediction plots based on the consensus values for 9 key variables of interest expressed as a function of the natural logarithm of patient age, whilst simultaneously taking into account the field strength of the MRI scanner. Solid lines represent model predictions, whereas shaded areas demarcate the 95%-prediction interval around these values (i.e., 95% of future measurements are expected to fall within these intervals). *R*^2^ values quantify the proportion of the total variability in the data the model accounts for, and *p*-values < 0.05 are taken to signify that the model performs better than an intercept-only model i.e., a straight, horizontal line, indicative of no growth). A spreadsheet to calculate values for the different variables for every patient’s age is provided as supplementary material. Abbreviations: trans = transverse, 3/3 = position 3 out of 3, supinf = superoinferior, lat = lateral

To complement the age-specific table of reference values, we further provide prediction plots obtained from the best overall performing growth model (Fig. 4), which allow generalization beyond the sample, rather than offering a bin-wise description of (subsets of) the data. AIC model comparisons revealed that the growth curve expressing the diameter of AVP structures as a linear function of the natural logarithm of patient age and MRI field strength [a + b_1_ × ln(Age) + b_2_ × MRI field strength], performed best for six out of nine variables (mean ΔAIC = − 2.80, range = − 9.98 – 2.66). For the diameter of the sheath 3/3 (ΔAIC = 1.27), as well as the lateral superoinferior (ΔAIC = 2.24) and the transverse diameter of the optic chiasm (ΔAIC = 2.66), another model performed marginally better. For reasons of biology (see Discussion) and parsimony, however, we depict predictions from the overall preferred model only. No statistically significant growth was apparent in the maximum retrobulbar diameter of the sheath (b_1_ ± SE = 0.07 ± 0.09, *p* = 0.442), nor for the lateral superoinferior diameters of the chiasm (b_1_ ± SE = 0.06 ± 0.04, *p* = 0.132). Moreover, size estimates for all AVP structures were consistently higher if a 1.5 T scanner was used than if a 3.0 T scanner was used (red line consistently above blue line in Fig. 4), although this difference did not achieve statistical significance in the transverse diameter of the globe (b_2_ ± SE = − 0.30 ± 0.20, *p* = 0.140), and the sheath 3.3 (b_2_ ± SE = − 0.04 ± 0.14, *p* = 0.802).

**Fig. 5:**
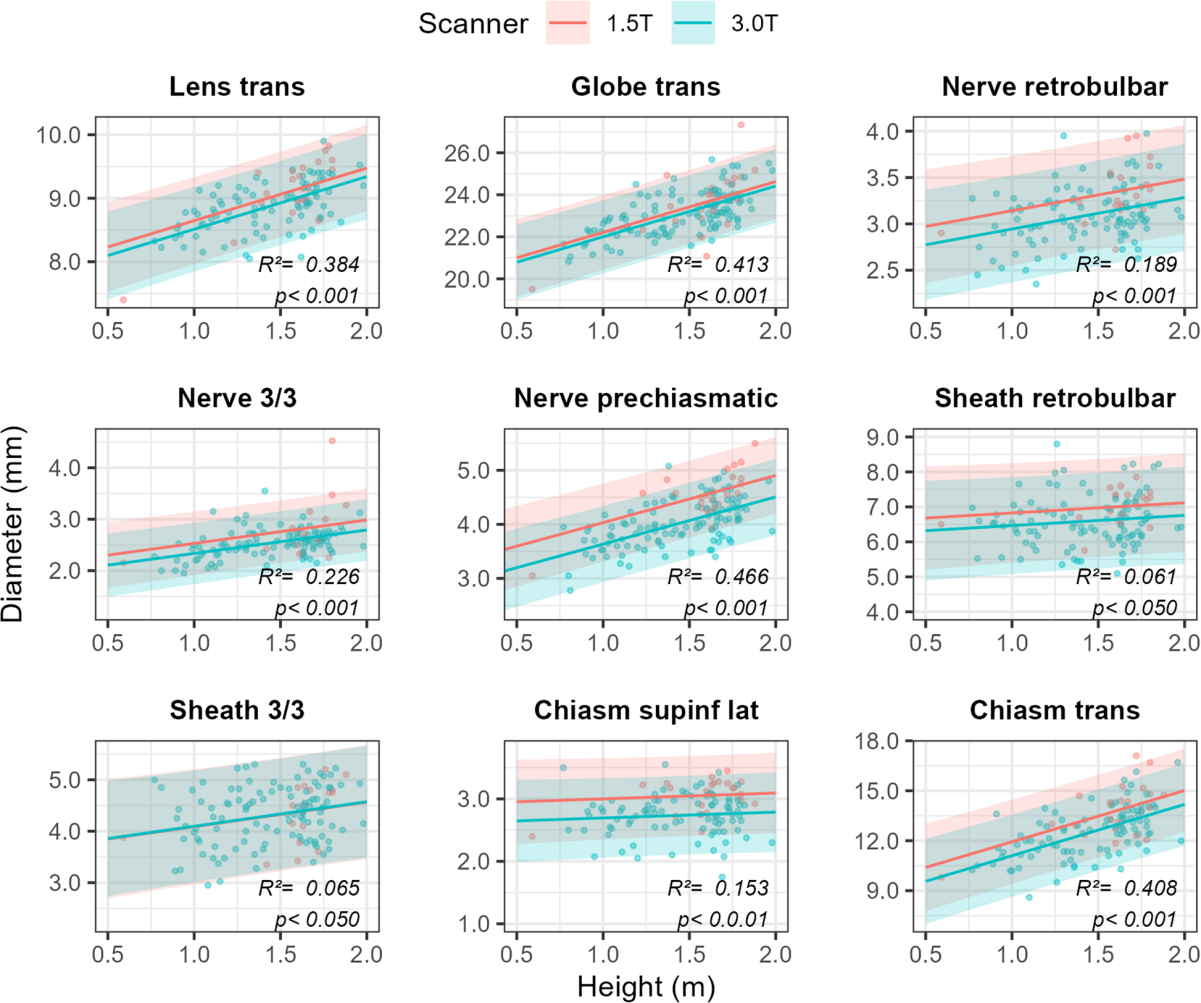
Prediction plots of consensus values for 9 key variables of interest expressed as a function of patient height, whilst simultaneously taking into account the field strength of the MRI scanner. Solid lines represent model predictions, whereas the shaded areas demarcate the 95%-prediction interval around these values (i.e., 95% of future measurements are expected to fall within these intervals). *R*^2^ values quantify the proportion of the total variability in the data the model accounts for, and *p*-values < 0.05 are taken to signify that the model performs better than an intercept-only model (i.e., a straight, horizontal line, indicative of no growth). A spreadsheet to calculate values for the different variables for every patient’s height is provided as supplementary material. Abbreviations: trans = transverse, 3/3 = position 3 out of 3, supinf = superoinferior, lat = lateral

In addition, prediction plots in which the 9 continuous variables of primary interest are expressed as a function of patient height (Fig. 5) were also created. In contrast to modelling the relationship with age, no compelling mechanistic reasons exist to consider curvilinear or non-linear relationships, and only predictions from a simple linear model are presented. These plots demonstrate the predicted modal range of expected values, given patient height, instead of age. The within-sample model performance (quantified by *R*^2^ values) is very similar to that of the growth models presented in the previous section, and in fact, the height model seems to (marginally) perform better for seven out of nine variables. Echoing our previous findings from the growth model, no statistically significant association between patient height and AVP structure size was apparent for the maximum retrobulbar diameter of the sheath (b_1_ ± SE = 0.29 ± 0.22, *p* = 0.189), or the lateral superoinferior diameter of the chiasm (b_1_ ± SE = 0.09 ± 0.11, *p* = 0.378). Also, diameters were consistently higher if a 1.5 T scanner was used than if a 3.0 T scanner was used (red line consistently above blue line in Fig. 5), and again this difference was not statistically significant for the transverse diameter of the globe (b_2_ ± SE = − 0.21 ± 0.19, p = 0.140), and the sheath 3.3 (b_2_ ± SE = − 0.01 ± 0.15, *p* = 0.954).

**Fig. 6:**
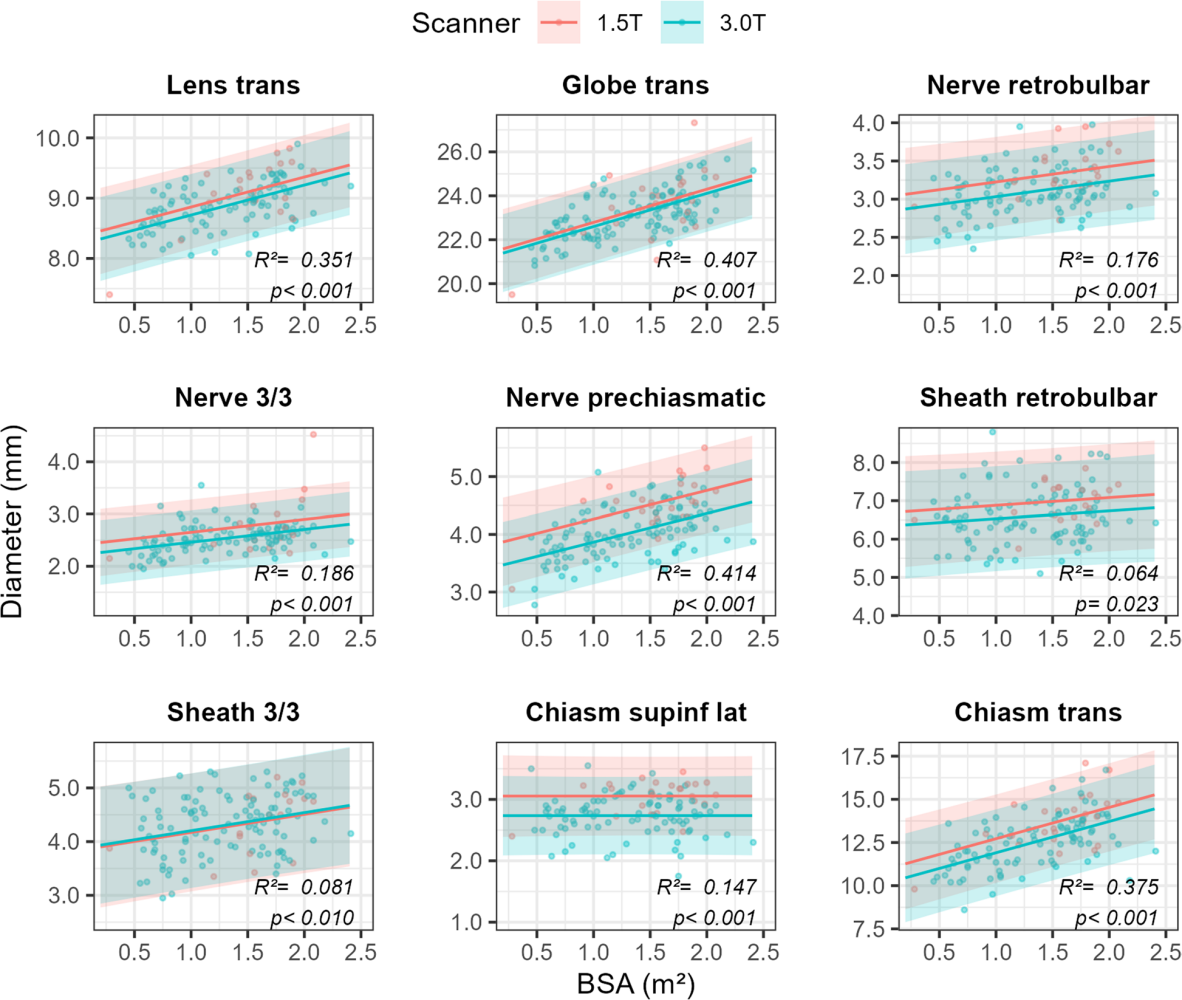
Prediction plots of consensus values for 9 key variables of interest expressed as a function of patient BSA, whilst simultaneously taking into account the field strength of the MRI scanner. Solid lines represent model predictions, whereas the shaded areas demarcate the 95%-prediction interval around these values (i.e., 95% of future measurements are expected to fall within these intervals). *R*^2^ values quantify the proportion of the total variability in the data the model accounts for, and *p*-values < 0.05 are taken to signify that the model performs better than an intercept-only model (i.e., a straight, horizontal line, indicative of no growth). Abbreviations: trans = transverse, 3/3 = position 3 out of 3, supinf = superoinferior, lat = lateral

Lastly, and analogous to the prediction plots generated for patient height, we provide graphs obtained from a series of linear models that predict the size of the 9 AVP structures of key interest as a function of patient BSA, whilst taking the effect of MRI field strength into account (Fig. 6). As was the case with the height models, within-sample performance (i.e. in terms of their *R*2 values) of the BSA models is on par with that of the original growth models, although the latter do perform slightly better on 7 out of 9. Maximum retrobulbar diameter of the sheath (b1 ± SE = 0.20 ± 0.14, *p* = 0.154) and diameter of the lateral superoinferior chiasm (b1 ± SE = 0.00 ± 0.07, *p* = 0.993) were not significantly associated with patient BSA. Moreover, MRI field strength did not significantly affect the values of the transverse diameter of the lens (b2 ± SE = 0.13 ± 0.08, *p* = 0.113), globe (b2 ± SE = 0.19 ± 0.20, *p* = 0.346), and sheath 3.3 (b2 ± SE = 0.03 ± 0.14, *p* = 0.822).

In addition to these prediction plots, we have created an “interactive” spreadsheet in an Excel Workbook to allow users to calculate the visualized normative values for the 9 AVP-structures of key interest themselves (Supplementary Material). The spreadsheet also takes the field strengths (1.5 T, 3 T) into account.

### Optic disc morphology and optic nerve tortuosity

Pooling all measurements across age bins and sides, 82% of children were scored to have a flat optic disk, 15% an excavation and 3% a protrusion. Tortuosity of the optic nerve occurred in 7% of children.

## Discussion

The normal size of anatomical structures, including the AVP, changes with growth. In addition, various pathologies may affect the size of AVP structures in children and adolescents [2, 3, 5]. Therefore, normative data can be very useful in differentiating normality from pathology. The AVP consists of small structures, especially in children, which can make accurate and reproducible measurements difficult. High-resolution 3D MRI sequences can be helpful in measuring these structures by enabling reformatting in any orientation allowing assessment of structures that do not follow the standard image planes. In recent years, optimized 3D T2w FSE sequences, which allow for single-slab 3D imaging of sizable volumes in an acceptable acquisition time have become available [17].

There are few articles in the literature regarding MRI normative data of AVP structure sizes in children, all of them using standard turbo spin echo sequences in standard planes for measurement [5, 6, 8, 9]. To our knowledge, there are no studies using high-resolution 3D MRI sequences to establish normative data of AVP structures in children and adolescents. In our study, measurements of globe, optic nerve, nerve sheath and optic chiasm diameters were all obtained on a 3D T2w FSE sequence using MPR to be able to measure each maximum diameter adjusted to the axis of the respective anatomical structure. This allowed for diameter measurements regardless of eye/lens position, optic nerve tortuosity or steepness of the intracranial optic nerve segment and optic chiasm.

Mncube et al. [11] showed that the optic chiasm transverse diameter is the most reliable measurement of the anterior visual pathway in an adult population as it is the largest structure and therefore easiest to measure. Our study supports this finding for children, as measurements of the optic chiasm transverse diameter have one of the highest ICC (left: 0.932, right: 0.960). A strong agreement was also demonstrated for the intracranial segment of the optic nerve (left: 0.754, right: 0.867), similar to what has been described by Mncube et al. [11]. Further reliable points of measurement of AVP structures are the transverse diameter of the globe (left: 0.937, right: 0.975) and the maximum diameter of the retrobulbar nerve sheath (left: 0.709, right: 0.903).

Another novelty explored in some detail in our study is formulation of biologically informed statistical models in addition to a reference table, explicitly incorporating what is known about the ontogeny of the human brain. Based on a sample of close to 3′500 brain weight values, Vannucci et al. [18] have described brain development as inherently non-linear, with growth being particularly pronounced during the first year of postnatal life, exhibiting a further stark increase between 1 and 5 years, and little growth thereafter. Therefore, in statistically modelling the diameters of the neuro-anatomical structures of interest in this study as a function of patient age, we endeavored to capture this empirically established developmental pattern by considering four competing mathematical equations commonly used to model biological growth. From a modelling perspective, it was unfortunate that the number of patients in the first year of life, when growth is most pronounced, was modest, creating some uncertainty in our model predictions at this age. However, in our experience MR examination at this age is very often pathological. This is supported by the fact, that during the time period of data acquisition for this study, a total of 116 MR examinations of the head were performed in children within the age bin of 0–3 years, and of these 103 (89%) were pathological. Nevertheless, based on a sample of 145 patients we could restore the biological (i.e., non-linear) growth curve, given that our preferred model (expressing the size of neuro-anatomical structures as a function of the natural logarithm of patient age) consistently outperformed a simpler (yet biologically unrealistic) model that expressed the relationship between structure size and patient age as a straight line. In addition, we found that age and height are approximately equal in predicting the dimensions of the different AVP structures and that patient height may offer the most reliable characteristic from which to infer reference values for the sizes of AVP structures. No statistically significant growth could be established for the maximum retrobulbar diameter of the sheath, nor for the lateral diameter of the chiasm.

We also found that foveal protrusion is seen in about 3% of normal pediatric head MRI. This should be taken into consideration when looking for findings of intracranial hypertension. In 15% we found excavation of the optic disc which may also occur in pathologies such as congenital coloboma (sporadic, familial, or syndromic, e.g., CHARGE syndrome) or morning glory syndrome [19–22]. In 7% we found a tortuous course of the optic nerve which can also be found in Neurofibromatosis type 1 [23, 24].

Lastly, when we corrected the normative values for the influence of MRI field strength, we found that most diameters were consistently higher with 1.5 T scanners compared to 3 T scanners. This may be attributed to a combination of different factors including slightly different CSF-tissue contrast properties, slightly different voxel dimensions and a slight difference in geometric distortions.

This study has some limitations. Firstly, it could be possible that at the time of study inclusion, a few participants may have had an undiagnosed subclinical (systemic/syndromic) disorder that may influence the size of the AVP structures, especially in the case of neonates. This problem could be addressed by a long-term follow-up (clinically and/or radiologically) and excluding patients who were later diagnosed with a syndromic or systemic disease. Secondly, due to the relatively small study population and the relatively large frequency of pathologies in the first years of life, children in the first years of life are underrepresented. Further subdivision of the patients from 0 to 3 years was therefore not appropriate. By including prediction plots obtained from a growth model, which does not divide patients into (unnatural/artificial) groups, we attempted to address this problem. Thirdly, we did not investigate the propensity of the 3D T2w FSE sequence to motion artefacts since this was not the aim of this study, but we excluded images with insufficient quality (e.g., due to motion artifacts). Fourthly, we did not use fat suppression, which could possibly increase the measurement accuracy. Fifthly, we did not separate the table of reference values by magnetic field strengths (1.5 T, 3 T) because the proportion of patients examined with 1.5 T was too small to create a separate table of reference values with age, size and BSA subgroups. However, we provide prediction plots (Figs. 4, 5 and 6) and an Excel spreadsheet (Supplementary Material) that take into account the field strength of the scanner.

## Conclusions

This study has established detailed charts of reference values and prediction plots using multiplanar reformations of 3D T2w FSE MR images allowing for axis-corrected measurements of anatomic structures along the anterior visual pathway in children. We have created a spreadsheet for users to calculate predicted values for patient age and height.

Measurements of the transverse diameter of the globe, the maximum diameter of the retrobulbar optic nerve sheath, the intracranial segment of the optic nerve and the chiasm transverse diameter were considered the most reliable measurements.

## Supplementary Information

**Additional file 1: Table S1.** Normative values (and associated 95%-prediction intervals) for the different AVP structures as predicted by the growth models developed and visualized (Fig. 4) in the main text of: Markart et al. 2022 “Pediatric reference values of anterior visual pathway structures measured with axis-correction on high-resolution 3D T2 fast spin echo sequences”. **Table S2.** Normative values (and associated 95%-prediction intervals) for the different AVP structures as predicted by the models developed and visualized (Fig. 5) in the main text of: Markart et al. 2022 “Pediatric reference values of anterior visual pathway structures measured with axis-correction on high-resolution 3D T2 fast spin echo sequences”.

## Data Availability

The datasets used and/or analyzed during the current study are available from the corresponding author on reasonable request.

## Abbreviations

3D: Three dimensional
AIC: Akaike information criterion
Antpost: Anteroposterior
AVP: Anterior visual pathway
BSA: Body surface area
CI: Confidence interval
CSF: Cerebrospinal fluid
CT: Computed tomography
FOV: Field of view
FSE: Fast Spin Echo
ICC: Intraclass correlation coefficient
IQR: Interquartile range
Lat: Lateral
MR: Magnetic resonance
MRI: Magnetic resonance imaging
MPR: Multiplanar reformation
PACS: Picture archiving and communication system
Supinf: Superoinferior
T2w: T2 weighted
TE: Echo time
TR: Repetition time
Trans: Transverse
